# Early toxicity prediction using longitudinal cone-beam CT delta-radiomics in HPV-positive oropharyngeal cancer

**DOI:** 10.3389/fonc.2026.1830230

**Published:** 2026-05-21

**Authors:** Levent Sensoy, Benjamin J. Rich, Stuart E. Samuels, Nesrin Dogan, Rodrigo Delgadillo

**Affiliations:** Department of Radiation Oncology, University of Miami Miller School of Medicine, Miami, FL, United States

**Keywords:** cone-beam CT (CBCT), delta-radiomics, dysphagia (swallowing disorder), oropharyngeal cancer (OPSCC), radiation therapy (radiotherapy), radiomics, supraglottic, toxicity prediction

## Abstract

**Introduction:**

Long-term survival in human papillomavirus–positive (HPV+) oropharyngeal squamous cell carcinoma (OPSCC) has shifted clinical focus toward minimizing treatment-related toxicities, particularly dysphagia, which significantly impacts quality of life. This study investigated whether longitudinal cone-beam computed tomography (CBCT)–based delta-radiomics of the supraglottic region can provide early indication of 6–12 month post-radiotherapy dysphagia.

**Methods:**

Forty-three patients with HPV+ OPSCC treated with definitive radiotherapy or chemoradiotherapy underwent daily CBCT imaging. Forty-three radiomic features were extracted from the supraglottic region of interest, volume-adjusted where appropriate, averaged weekly, and transformed using a ratio-to-baseline formulation anchored to the first treatment-day CBCT. Spearman correlation filtering (|ρ| > 0.8) reduced redundancy prior to Random Forest ranking using out-of-bag permutation importance. Logistic regression models were constructed using incrementally increasing feature subsets across individual weeks and grouped multi-week CBCT temporal windows. Model performance was evaluated using repeated random two-thirds resampling with 1000 iterations, and discrimination was quantified by the mean area under the receiver operating characteristic curve with 95% percentile confidence intervals.

**Results:**

The study cohort included 43 patients with HPV+ OPSCC treated with definitive radiotherapy. Predictive signal emerged early during treatment. The Weeks 1–4 model achieved a mean area under the curve of 0.779 (95% confidence interval: 0.714–0.812) using three features, outperforming the Week 2 model (0.728) and demonstrating comparable discrimination to the Weeks 1–6 model (0.795) with greater parsimony. Global skewness was consistently the highest-ranked predictor across temporal windows.

**Conclusion:**

Supraglottic CBCT-based delta-radiomics demonstrates an early signal associated with post-radiotherapy dysphagia in HPV+ OPSCC. A ratio-to-baseline longitudinal framework combined with weekly aggregation enabled robust modeling while preserving interpretability. Given the limited cohort size and absence of external validation, these findings should be interpreted as exploratory and hypothesis-generating.

## 1 Introduction

Human papillomavirus (HPV)-associated OPSCC is characterized by favorable prognosis, with reported 5-year survival rates exceeding 80% in the United States. Clinical attention has therefore increasingly shifted toward minimizing treatment-related toxicities in survivors (1–5). In HPV-positive (HPV+) OPSCC, long-term treatment effects such as xerostomia, dysgeusia, and particularly dysphagia remain important survivorship concerns. Dysphagia can persist for years post-radiation therapy (RT), significantly impacting nutritional status, quality of life, and overall survivorship (6–8). Radiomics may be useful in this regard due to its ability to aid in early detection of treatment-related changes and toxicity risk stratification.

Radiomics has emerged as a quantitative imaging biomarker strategy capable of noninvasively predicting treatment response and toxicity (9–12). In head and neck (H&N) cancer, most radiomics studies to date rely on static imaging—typically the planning computed tomography (CT)—to extract features that characterize tumor heterogeneity and surrounding tissue characteristics (13). However, a growing body of literature supports the concept of delta-radiomics, which leverages temporal changes in imaging features over the course of radiotherapy to capture treatment-induced biological effects (14–16). Early investigations have demonstrated the potential of delta-radiomics for outcomes prediction across multiple disease sites, including H&N, liver, and lung cancers (17–19).

Cone-beam CT (CBCT), routinely acquired daily or weekly during radiotherapy for patient setup verification, provides a unique opportunity to longitudinally monitor anatomical and structural changes without additional imaging burden. Despite this advantage, its use for radiomics remains technically challenging due to limitations in image quality, increased noise, scatter artifacts, and lack of standardized acquisition protocols (20–22). Nevertheless, a growing body of work has explored the use of CBCT-based radiomics and delta-radiomics for outcome prediction in head and neck cancer. Marcu et al. reviewed the emerging role of delta-radiomics in head and neck oncology and highlighted the potential of longitudinal imaging features for predicting treatment response and normal-tissue toxicity (23). van Dijk et al. demonstrated that longitudinal CT-based delta-radiomic features acquired during radiotherapy improved prediction of late xerostomia by capturing treatment-related temporal feature evolution (24). Sellami et al. analyzed CBCT-derived radiomic features in head and neck squamous cell carcinoma and showed that longitudinal CBCT radiomics combined with clinical variables could accurately predict treatment response (25). Morgan et al. further demonstrated that combining baseline CT features with intra-treatment CBCT radiomic changes improved prediction of local failure using interpretable machine-learning models (26). These studies have primarily focused on predicting treatment response and oncologic outcomes. In contrast, the present study extends the delta-radiomics framework to toxicity prediction, specifically targeting radiation-induced swallowing dysfunction rather than tumor prognosis.

In this study, we developed and evaluated a longitudinal CBCT-based delta-radiomics modeling framework to predict post-treatment dysphagia in patients with HPV+ OPSCC treated with definitive radiotherapy. To our knowledge, this is among the first investigations to leverage daily CBCT-derived delta-radiomic features for toxicity prediction in HPV+ OPSCC.

## 2 Materials and methods

### 2.1 Patient cohort and outcome assessment

This retrospective study included 43 patients with biopsy-confirmed HPV-positive oropharyngeal squamous cell carcinoma (HPV+ OPSCC) treated between 2018 and 2022 under an institutional review board–approved protocol. Inclusion criteria were: (1) histologically confirmed HPV+ OPSCC, (2) treatment with definitive radiotherapy to 70 Gy in 35 fractions, (3) availability of planning CT and daily CBCT imaging, and (4) documented swallowing assessment at 6–12 months following radiotherapy completion.

Dysphagia was assessed 6–12 months after radiotherapy, when acute treatment-related inflammation was expected to have resolved and chronic changes could be evaluated. Severity was categorized as none, mild, moderate, or severe, with speech pathology confirmation when available. For modeling purposes, none and mild were grouped as “tolerable,” and moderate and severe were grouped as “significant”.

### 2.2 Imaging acquisition and region of interest delineation

All patients were immobilized using a thermoplastic mask and treated with volumetric modulated arc therapy (VMAT) on Varian linear accelerators (Varian Medical Systems, Palo Alto, CA). Daily CBCT images were acquired prior to each treatment fraction using onboard imaging systems, yielding serial three-dimensional datasets for longitudinal analysis. Imaging was performed across multiple linear accelerator platforms, including TrueBeam-A (67%), TrueBeam-B (21%), and Trilogy (12%). TrueBeam-A acquisitions used iterative reconstruction, whereas TrueBeam-B and Trilogy acquisitions used standard reconstruction. Pixel spacing ranged from 0.5–0.6 mm and slice thickness from 2.0–2.3 mm, with broad variability in exposure parameters across systems. CBCT acquisition parameters are summarized in **Table 1**.

**Table 1:** CBCT acquisition parameters and reconstruction characteristics across the 43-patient cohort, with 35 treatment fractions per patient.

Linear accelerator model	TrueBeam-A	TrueBeam-B	Trilogy
% of all scans (N = 43 × 35)	67%	21%	12%
Reconstruction method	iterative	standard	standard
Width & Height (pixels)	512	506.5 ± 26.1 (384-512)	429.1 ± 61.3 (384-512)
Pixel Spacing (mm)	0.5	0.5	0.6 ± 0.1 (0.5-0.7)
Slice Thickness (mm)	2.0	2.0	2.3 ± 0.2 (2.0-2.5)
Field of View (mm)	262	261.5 ± 2.4 (250-262)	254.2 ± 5.7 (250-262)
Tube Voltage (kVp)	100	100	102.4 ± 7.3 (100-125)
Tube Current (mA)	15	17.6 ± 12.8 (15-80)	41.4 ± 29.3 (10-80)
Exposure (mAs)	150.2 ± 0.9 (150-151)	150.9 ± 0.4 (150-151)	192.5 ± 150.9 (69-728)

Values reflect platform-dependent variability in reconstruction algorithm, image dimensions, voxel geometry, field of view, and exposure parameters. Parameters with variable values are reported as mean ± standard deviation (range), where applicable.

The supraglottic region of interest (ROI) was delineated on the planning CT by an experienced H&N radiation oncologist. Planning contours were propagated to each daily CBCT using deformable image registration (DIR) implemented in MIM Maestro (v7.4.3; MIM Software, Cleveland, OH, USA) and subsequently visually reviewed (27). Manual adjustments were performed when necessary, by the same radiation oncologist to ensure anatomical accuracy and contour consistency across treatment fractions.

### 2.3 Radiomic feature extraction

Radiomic features were extracted from the supraglottic ROI on each daily CBCT using MATLAB (MathWorks, Natick, MA), in accordance with Image Biomarker Standardization Initiative (IBSI)–compliant definitions. Prior to feature extraction, images were resampled to isotropic voxel spacing to ensure spatial consistency and minimize interpolation-related variability.

A total of 43 features were extracted per image, including 42 IBSI-defined features derived from first-order intensity statistics and higher-order texture matrices, including gray-level co-occurrence matrix (GLCM), gray-level run-length matrix (GLRLM), gray-level size zone matrix (GLSZM), and neighborhood gray-tone difference matrix (NGTDM) features. Supraglottic volume was included as an additional geometric descriptor. The complete list of IBSI-compliant radiomic features and corresponding reference identifiers is provided in **Supplementary Table S1**.

### 2.4 Volume adjustment of selected features

Radiomic feature values are known to scale with region-of-interest volume, potentially introducing confounding effects when anatomical size varies across patients or over time. To mitigate this effect, selected size-dependent features were replaced with volume-adjusted variants using previously described normalization frameworks.

Volume normalization was applied to GLRLM Run Length Non-Uniformity (RLN), GLRLM Gray Level Non-Uniformity (GLN), GLSZM Gray Level Non-Uniformity (GLN), GLSZM Zone Size Non-Uniformity (ZSN), and NGTDM Strength (28). In addition, a feature–volume normalization approach was applied to NGTDM Busyness and NGTDM Coarseness (29). Supraglottic volume was retained as an independent feature to allow direct modeling of anatomical size effects.

### 2.5 Weekly aggregation and delta-radiomics computation

To reduce day-to-day variability and reflect clinically meaningful treatment intervals, daily radiomic features were averaged over fixed five-fraction blocks corresponding to treatment weeks.

For a given feature, the weekly mean value for week was computed as:


$$\bar{X_{\text{i},w}}=\frac{1}{N_{w}}\sum_{f=1}^{N_{w}}X_{\text{i},w,f}$$

where *N_w_* denotes the number of fractions within week *w*, and X_i,w,f_ represents the value of feature *i* at fraction *f* of week *w*.

Delta features were defined relative to the CBCT preceding the first treatment fraction (baseline). The week-specific delta was calculated as:


$${\text{Δ}}_{\text{i},w}=\frac{\bar{X_{\text{i},w}}}{X_{\text{i},1}}$$

where *X_i_*_,1_ denotes the feature value from fraction 1. The first treatment-day CBCT was used as the baseline reference to ensure consistency with the imaging modality and acquisition conditions used throughout treatment. While this differs from conventional pre-treatment planning CT–based baselines, it avoids cross-modality variability and enables internally consistent longitudinal delta-radiomic characterization.

For grouped temporal windows, weekly means were first averaged across selected weeks:


$$\bar{X_{\text{i},W}}=\frac{1}{\left|W\right|}\sum_{w\in W}\bar{X_{\text{i},w}}$$

where *W* denotes the selected set of treatment weeks and |*W*| represents the number of weeks in that grouping. The grouped delta was then computed as:


$${\text{Δ}}_{\text{i},W}=\frac{\bar{X_{\text{i},W}}}{X_{\text{i},1}}$$

This ratio-to-baseline formulation was applied consistently across all temporal intervals.

### 2.6 Correlation-based feature reduction

To mitigate multicollinearity, a Spearman rank correlation matrix was computed across patients for each temporal interval. Features with an absolute pairwise correlation coefficient exceeding 0.8 were considered redundant. From each highly correlated feature pair, one feature was removed according to a predefined filtering criterion to preserve feature independence. The resulting reduced feature set was subsequently used for feature ranking and model development for each temporal configuration.

### 2.7 Random forest feature ranking

Feature ranking was performed using a Random Forest classifier implemented in MATLAB using the *TreeBagger* function with 500 decision trees. Individual trees were constructed using the Gini impurity [Gini’s diversity index (30)] as the node-splitting criterion. Feature importance was quantified using out-of-bag permutation importance (OOB permuted predictor delta error), rather than impurity-based importance metrics. Feature ranking was conducted independently for each temporal interval and grouped-week configuration.

### 2.8 Logistic regression modeling and feature count optimization

Logistic regression models were constructed using MATLAB’s *fitglm* function with a binomial distribution and logit link. Incremental feature subsets (K = number of features) were evaluated by selecting the top-ranked features with positive importance, where K ranged from 1 to a maximum of 7 (or fewer if limited by the number of positively ranked features). This approach enabled identification of the optimal model dimensionality for each feature aggregation time window.

### 2.9 Model performance evaluation

**Figure 1:**
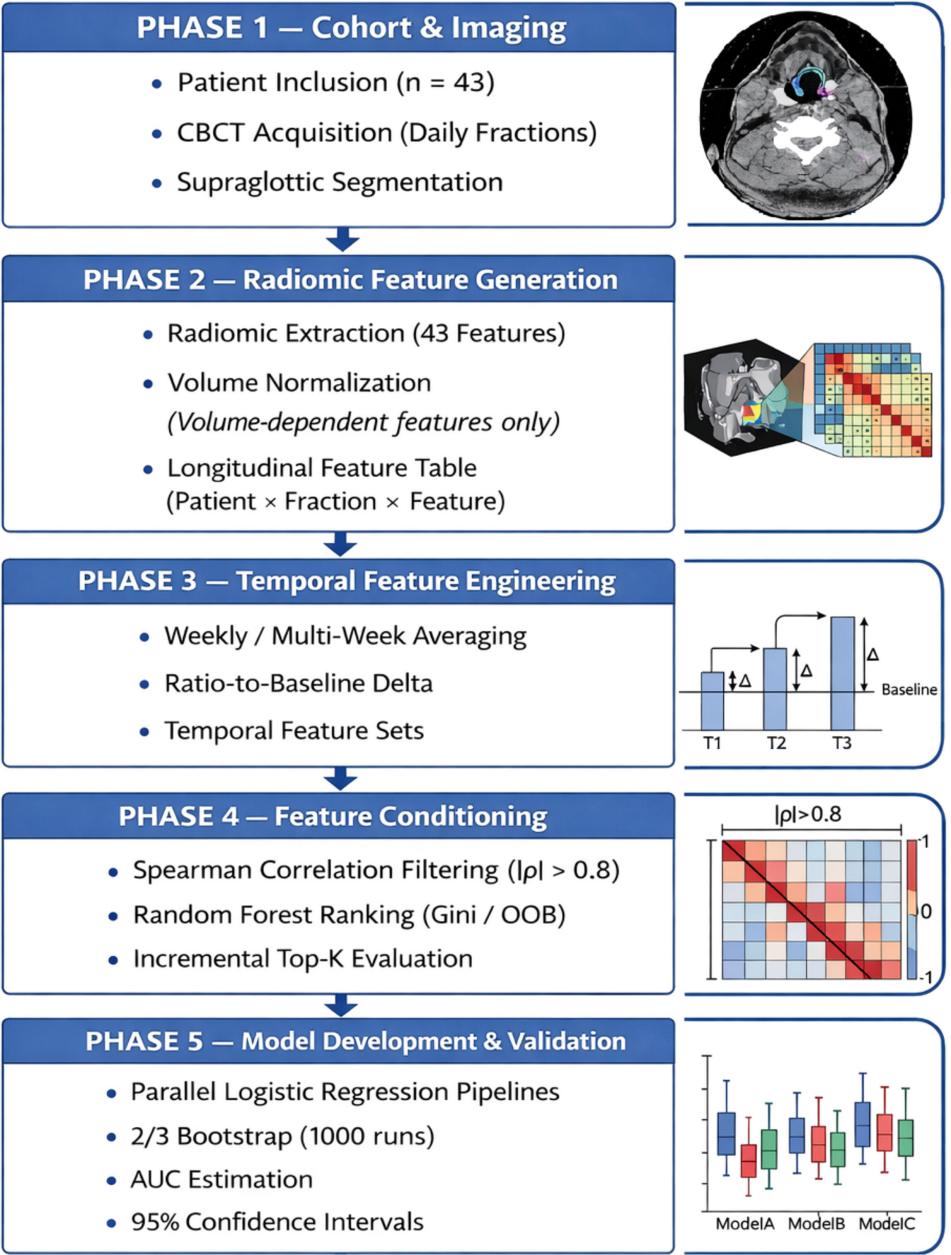
Workflow of the CBCT delta-radiomics modeling pipeline. Daily CBCT features were aggregated into weekly means and transformed into ratio-to-baseline deltas (week/baseline). Redundant features were removed using Spearman correlation filtering (|ρ| > 0.8). Random Forest models were constructed using the Gini impurity (Gini’s diversity index) as the node-splitting criterion, and feature ranking was performed using out-of-bag (OOB) permutation importance. Logistic regression models were evaluated using repeated random two-thirds resampling (1000 iterations). AUC values are reported as mean with 95% percentile-based confidence intervals (2.5th–97.5th).

Model performance was evaluated using repeated random two-thirds resampling. In each iteration, two-thirds of the cohort were randomly selected for model fitting, and predictions were generated for all patients. The AUC was computed for each iteration. Resampling was performed without explicit class stratification; however, the use of repeated random sampling and AUC-based evaluation mitigates sensitivity to class imbalance. Because discrimination was summarized using the area under the receiver operating characteristic curve (AUC), a threshold-independent metric that is less sensitive to class prevalence than accuracy-based measures, no class reweighting or synthetic oversampling was applied. The mean AUC and 95% percentile-based confidence intervals were derived from the empirical resampling distribution using the 2.5th and 97.5th percentiles. A total of 1000 resampling iterations were performed for each model configuration. Because feature selection steps were performed prior to resampling, the resulting performance estimates should be interpreted as internal measures of model discrimination rather than unbiased estimates of generalizable performance. A schematic overview of the full modeling workflow is provided in **Figure 1**.

## 3 Results

### 3.1 Cohort and imaging characteristics

A total of 43 HPV+ OPSCC patients were included in the final analysis. At 6–12 months post-radiotherapy, 28/43 patients (65.1%) were classified as having tolerable dysphagia and 15/43 (34.9%) as having significant dysphagia. All patients underwent daily CBCT imaging with supraglottic segmentation and longitudinal extraction of 43 radiomic features per fraction. Patient and disease characteristics are summarized in **Table 2**.

**Table 2:** Patient and disease characteristics of the 43 HPV-positive OPSCC patients included in the study cohort.

Characteristics		n (%)
Total patients		43
Age (in years) – median		59.5
Sex		
	Female	9 (20.9)
	Male	34 (79.1)
Oropharyngeal subsite		
	Tonsil	23 (53.5)
	Base of tongue	20 (46.5)
	Posterior wharyngeal Wall	0 (0)
	Soft palate	0 (0)
HPV status		
	HPV-positive	43 (100.0)
	HPV-negative	0 (0)
AJCC 8^th^ ed. Cancer stage		
	I	23 (53.5)
	II	17 (39.5)
	III	3 (7.0)
Tumor stage		
	1	11 (25.6)
	2	21 (48.8)
	3	9 (20.9)
	4	2 (4.7)
Nodal stage		
	0	3 (7.0)
	1	27 (62.8)
	2	12 (27.9)
	3	1 (2.3)
Concurrent systemic therapy		
	None	5 (11.6)
	Chemotherapy	37 (86.0)
	Other	1 (2.3)

### 3.2 Correlation structure and feature conditioning

Spearman correlation analysis of the 43 ratio-to-baseline delta features demonstrated substantial redundancy across texture families (**Supplementary Figure S1**). Clustered correlation blocks were observed within GLCM, GLRLM, and GLSZM feature groups, with multiple feature pairs exceeding the prespecified threshold of |ρ| > 0.8.

Correlation filtering was applied independently within each temporal grouping prior to Random Forest ranking. This reduced the initial 43-feature set to 34 features for the Week 2 model, 31 features for the Weeks 1–4 model, and 32 features for the Weeks 1–6 model. This preprocessing step mitigated multicollinearity while preserving representative predictors across radiomic families.

### 3.3 Random forest ranking and feature selection

Random Forest modeling (500 trees; out-of-bag permutation importance) was performed using the correlation-filtered feature sets for each temporal window. Predictor importance was quantified as the increase in out-of-bag classification error following random permutation of each feature.

**Figure 2:**
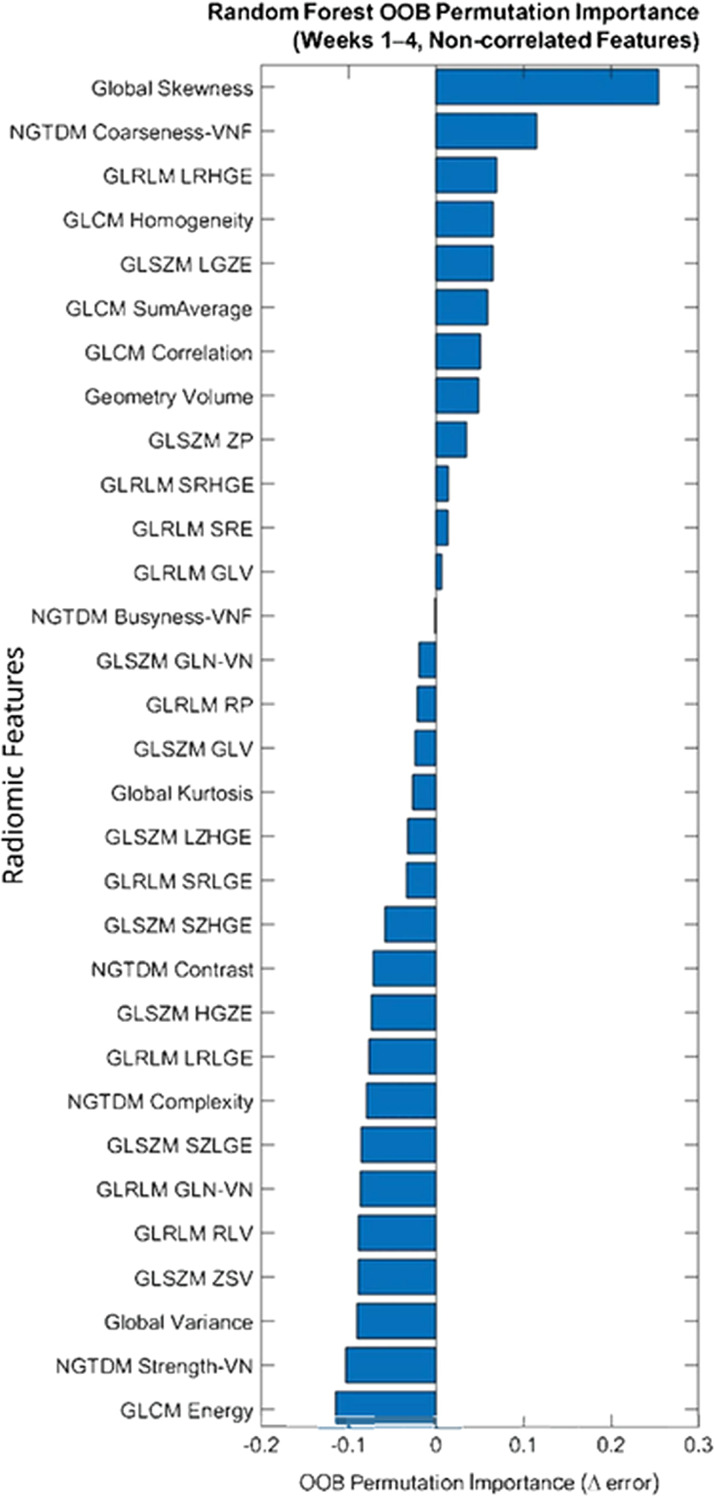
Random Forest predictor ranking for the weeks 1–4 temporal aggregation model using out-of-bag (OOB) permutation importance. Bars represent the increase in OOB classification error (Δ error) following feature permutation.

Global Skewness ranked highest by OOB permuted importance across all temporal windows (**Figure 2** for Weeks 1–4; **Supplementary Figures S2** and **S3** for Week 2 and Weeks 1–6, respectively). Although the magnitude of importance varied across groupings, the relative ranking of key features remained stable.

For Week 2 grouping, the optimal model consisted of K = 4 features. The Weeks 1–4 and Weeks 1–6 groupings achieved optimal performance with K = 3 and K = 7 features, respectively. The selected feature subsets and corresponding out-of-bag (OOB) permuted importance values are provided in **Table 3**. Global Skewness was retained across all temporal groupings, suggesting a consistent predictive contribution. The optimal feature dimensionality differed across temporal configurations, with the Weeks 1–6 model requiring a larger subset of features to achieve maximal performance.

**Table 3:** Final selected radiomic features for each optimized temporal aggregation model.

Temporal window (number of features)	Selected features (OOB permuted importance)
Week 2 (4)	Global Skewness (0.205); GLRLM LRE (0.066); Geometry Volume (0.040); GLRLM RLN-VN (0.029)
Weeks 1–4 (3)	Global Skewness (0.270); NGTDM Coarseness-VNF (0.099); GLCM Homogeneity (0.094)
Weeks 1–6 (7)	Global Skewness (0.287); GLSZM LGZE (0.059); GLRLM SRHGE (0.047); GLRLM RLV (0.028); GLCM Homogeneity (0.022); GLRLM LGRE (0.021); GLRLM SRLGE (0.019)

Features are listed in descending order of Random Forest out-of-bag permuted predictor importance. Importance values represent the increase in out-of-bag classification error following predictor permutation.

### 3.4 Logistic regression performance across temporal windows

Model discrimination was evaluated using repeated two-thirds resampling with 1000 iterations. AUC values are reported as mean (95% percentile CI, 2.5th–97.5th).

**Figure 3:**
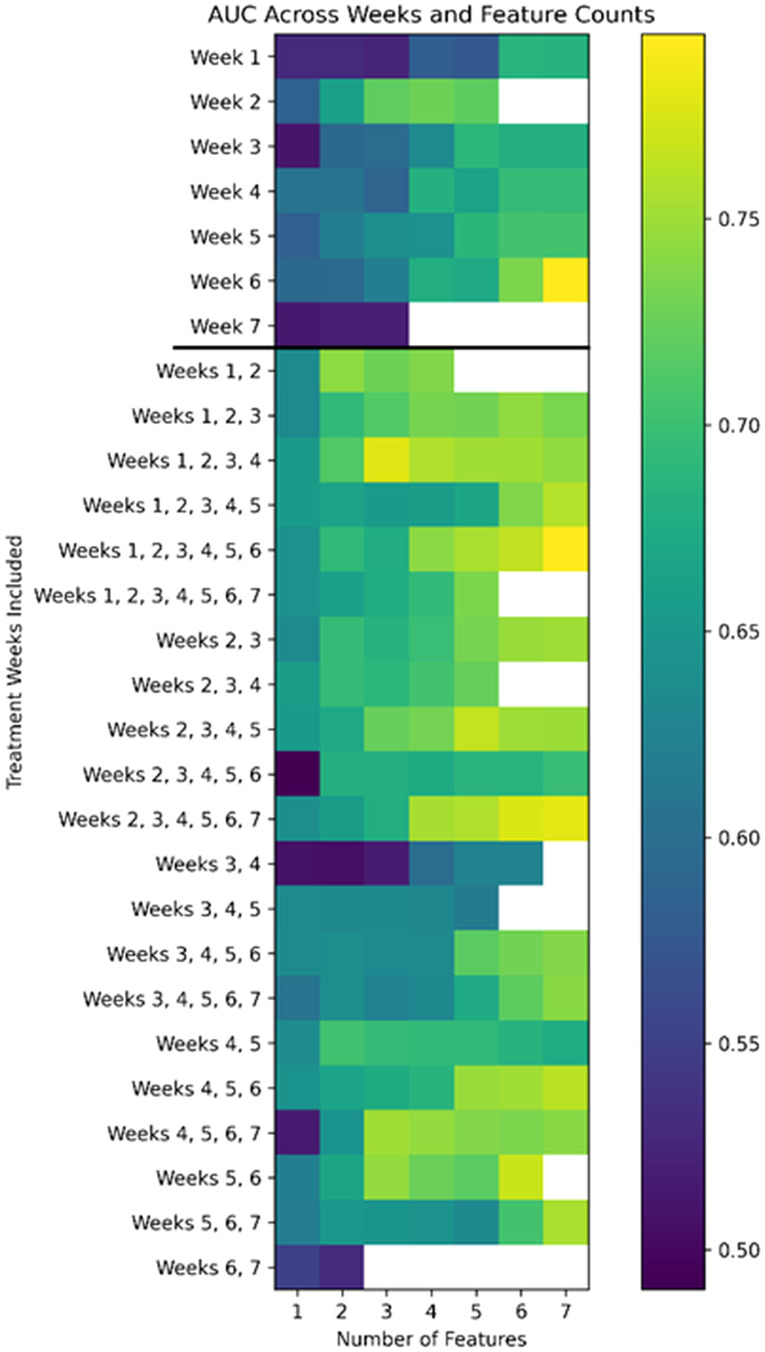
Heatmap of mean AUC across temporal aggregation windows and feature counts. Performance metrics were derived from 1000 repeated random two-thirds resampling iterations. Warmer colors indicate higher mean AUC values.

The comprehensive performance landscape across all temporal windows and feature counts is shown in **Figure 3**. Discriminatory performance increased with feature count and subsequently plateaued for each temporal grouping.

The Weeks 1–4 model achieved a mean AUC of 0.779 (95% CI: 0.714–0.812) using three features. The Weeks 1–6 model achieved a mean AUC of 0.795 (95% CI: 0.703–0.840) using seven features. The Week 2 model achieved a mean AUC of 0.728 (95% CI: 0.593–0.808) using four features.

**Figure 4:**
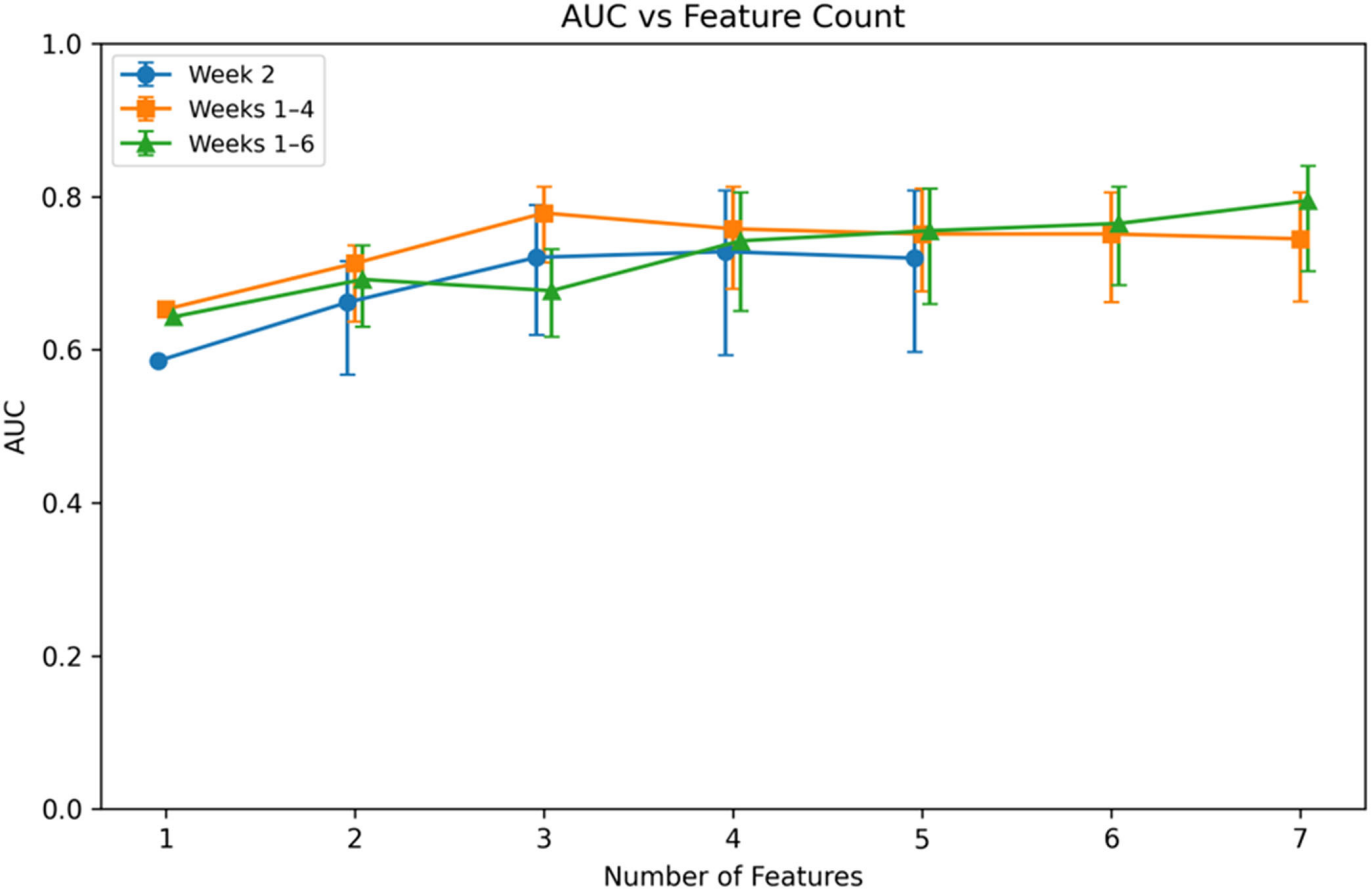
AUC versus feature count for week 2, weeks 1–4, and weeks 1–6 temporal models. Error bars represent 95% percentile-based confidence intervals (2.5th–97.5th) derived from 1000 repeated two-thirds resampling iterations. Curves are slightly offset along the x-axis for visual clarity.

Direct comparison of model evolution across feature counts is shown in **Figure 4**. Individual feature-count optimization trajectories for the Week 2 and Weeks 1–6 models are additionally provided in **Supplementary Figures S4**, **S5**, respectively. Aggregation through Weeks 1–4 improved discrimination relative to Week 2. Extension to Weeks 1–6 yielded a modest additional increase in mean AUC while requiring a larger feature subset.

**Figure 5:**
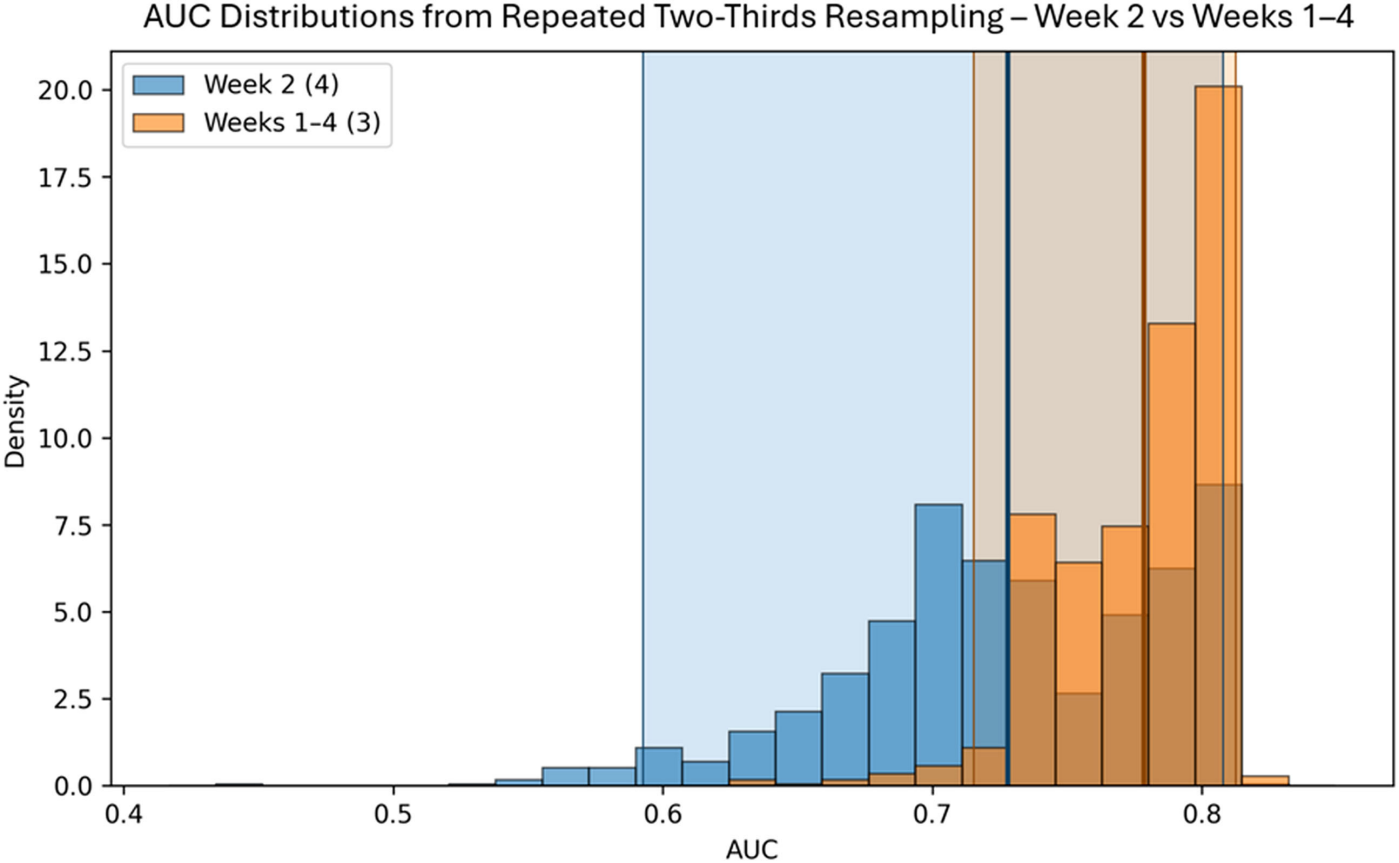
Distribution of AUC values from 1000 repeated two-thirds resampling iterations for the optimal Week 2 (4 features) and Weeks 1–4 (3 features) models. Histograms represent the empirical AUC distribution across iterations. Thick vertical lines denote mean AUC values, and shaded regions indicate the 95% percentile-based confidence intervals (2.5th–97.5th).

Resampling AUC distributions derived from 1000 two-thirds resampling iterations are shown in **Figure 5** for Week 2 and Weeks 1–4. The Weeks 1–4 distribution demonstrates a rightward shift relative to Week 2, with overlapping but distinct percentile intervals.

Threshold-based performance metrics corresponding to the final models are provided in the **Supplementary Materials** (**Supplementary Table S2**) for completeness.

## 4 Discussion

### 4.1 Overview and principal findings

This study demonstrates that supraglottic CBCT delta-radiomics, defined using weekly averaging and a ratio-to-baseline framework anchored to the first treatment-day CBCT, can differentiate patients who develop post-radiotherapy dysphagia in HPV-positive oropharyngeal cancer. Predictive signal emerged early in treatment and strengthened over the first four weeks. Extension of the observation window to six weeks did not produce a meaningful improvement in model discrimination despite increased model dimensionality, with the Weeks 1–4 model providing a more parsimonious and temporally earlier predictive signal. Given improved parsimony, the Weeks 1–4 model is considered the primary model in this study, balancing predictive performance with model stability and clinical interpretability.

The observed temporal behavior suggests that radiomic evolution within supraglottic soft tissues captures radiographically meaningful acute-phase changes that precede clinically manifest swallowing dysfunction. Importantly, improvements in discrimination beyond Week 4 were modest and required a higher number of selected features (K = 7 for Weeks 1–6 versus K = 3 for Weeks 1–4), suggesting greater model complexity and reduced parsimony at later timepoints.

### 4.2 Biological interpretation of supraglottic radiomic change

The supraglottic region plays a central role in airway protection and swallowing coordination. Radiation-induced dysphagia is multifactorial, involving mucosal inflammation, edema, neuromuscular dysfunction, and fibrosis of pharyngeal constrictors and laryngeal structures (31). Structural and functional alterations of pharyngeal constrictor muscles during and after radiotherapy have been systematically documented, supporting the biological plausibility of early tissue changes preceding clinical swallowing impairment (32).

Functional imaging studies further reinforce this concept. Mid-treatment perfusion alterations in pharyngeal constrictor muscles have been shown to correlate with subsequent dysphagia (33, 34). These findings align with the interpretation that early microvascular and structural changes within the swallowing apparatus precede clinically manifest toxicity.

The consistent prominence of Global Skewness across temporal windows suggests that asymmetry in intensity distribution—potentially reflecting evolving heterogeneity within supraglottic soft tissues—may be associated with early treatment-related tissue changes, although this interpretation remains hypothetical and requires further biological validation. Among the radiomic descriptors evaluated, Global Skewness consistently ranked highest in Random Forest importance across temporal windows, indicating that distributional asymmetry of voxel intensities may capture early microstructural heterogeneity associated with treatment-related tissue response.

The predominance of higher-order texture descriptors rather than purely geometric metrics further supports the hypothesis that micro-architectural alteration, rather than gross volume change, underlies the predictive signal.

Selective application of volume normalization was therefore methodologically appropriate. While certain radiomic features scale mathematically with region-of-interest volume and benefit from normalization, changes in supraglottic volume during radiotherapy may reflect treatment-related edema, which is biologically relevant and may contribute directly to swallowing dysfunction.

### 4.3 Temporal radiobiological considerations

The temporal pattern observed here aligns with established clinical onset patterns of radiation-associated dysphagia. Clinical analyses demonstrate that acute symptoms during the course of head and neck radiotherapy are strong predictors of chronic dysphagia, emphasizing the importance of early-treatment biological changes (35, 36).

The Week 2 window likely captures the onset of inflammatory and microvascular responses within supraglottic tissues, consistent with the cumulative inflammatory effects produced by repeated radiation fractions (37). Extending analysis to Weeks 1–4 consolidates this evolving signal within the acute-phase response period.

By contrast, Weeks 1–6 incorporate a broader mixture of treatment-related processes occurring during later phases of radiotherapy, including cumulative edema and mucosal injury associated with treatment-related dysphagia (38), treatment-related nutritional decline and weight loss (39), and progressive anatomical changes such as tumor shrinkage or weight loss that may influence adaptive replanning considerations (40).

Consistent with this broader mixture of biological and treatment-related effects, the Weeks 1–6 model required a larger feature subset to achieve maximal performance, suggesting a more diffuse predictive signal and reduced parsimony.

### 4.4 Choice of delta formulation

The operational definition of relative temporal change (“delta”) in delta-radiomics varies across studies, with some defining delta as a percentage or normalized difference relative to baseline and others using ratio-to-baseline formulations anchored to a pre-treatment or first-fraction scan (15, 17, 41). In the present study, longitudinal change was defined as a ratio to the first treatment-day CBCT baseline.

This approach was driven by statistical and interpretative considerations. The ratio formulation preserves proportional change and scale invariance across patients and aligns with a multiplicative model of tissue evolution during radiotherapy. Importantly, ratio avoids denominator-driven amplification inherent to normalized-difference formulations, which may become unstable when baseline values are small—a relevant consideration for CBCT-derived radiomic features.

### 4.5 Stability versus predictive relevance

Radiomic stability is frequently discussed as a prerequisite for biomarker validity. However, reproducibility does not necessarily equate to predictive informativeness. Radiomic feature repeatability and reproducibility are influenced by acquisition parameters, reconstruction algorithms, segmentation consistency, and preprocessing pipelines (42–45).

In the present cohort, most ratio-to-baseline delta features demonstrated favorable stability profiles, characterized by high intraclass correlation coefficient (ICC)-like indices and low within-patient variability (**Supplementary Figure S6**). A minority exhibited elevated variability, as reflected by mean within-patient coefficient of variation (**Supplementary Figure S7**). A minority exhibited elevated variability. Corresponding mean within-patient standard deviation profiles are shown in **Supplementary Figure S8**. Importantly, methodological analyses have emphasized that excluding features solely on the basis of reproducibility may discard biologically informative predictors (46, 47). The relationship between complementary stability measures is shown in **Supplementary Figure S9**. Accordingly, stability was treated as contextual evidence rather than a strict exclusion criterion. Correlation filtering (Spearman |ρ| > 0.8) reduced redundancy without enforcing temporal invariance, and Random Forest out-of-bag permutation importance prioritized predictive contribution.

### 4.6 Imaging heterogeneity and reconstruction effects

CBCT acquisition in this cohort was heterogeneous, including multiple linac platforms, wide mAs ranges, and mixed reconstruction techniques. Acquisition and reconstruction parameters are known to influence radiomic feature behavior and reproducibility (42–45). Although longitudinal anchoring to patient-specific baselines may mitigate absolute intensity scale sensitivity, reconstruction heterogeneity remains a limitation and warrants harmonization strategies in future studies. Importantly, this heterogeneity reflects routine clinical practice, where patients may undergo imaging on different systems over the course of treatment, thereby enhancing the pragmatic relevance of the present findings.

No explicit intensity normalization or harmonization strategy was applied in the current study, and therefore scanner-dependent effects cannot be fully excluded as contributors to the observed radiomic signal.

### 4.7 Methodological robustness and translational implications

Model discrimination was evaluated using repeated random two-thirds resampling with 1000 iterations, with AUC reported as the mean and 95% percentile-based confidence intervals (2.5th–97.5th). Although the outcome distribution was moderately imbalanced, discrimination was summarized using AUC, a threshold-independent metric that is less sensitive to class prevalence than accuracy-based measures. Feature dimensionality was constrained via Random Forest ranking and conservative correlation filtering, helping mitigate overfitting relative to cohort size**;** however, because feature selection was performed prior to resampling, the reported performance metrics should be interpreted as internal estimates and may be subject to optimistic bias.

Clinically, emergence of predictive signal early in the radiotherapy course suggests potential integration into radiotherapy workflows, enabling earlier supportive intervention or treatment modification (48).

### 4.8 Limitations and future directions

This study has several limitations, and should be interpreted as an exploratory, hypothesis-generating analysis rather than a validated predictive modeling study. The relatively small cohort size (n = 43), absence of external validation, and lack of explicit CBCT reconstruction harmonization limit generalizability and should be addressed in future multi-institutional investigations incorporating standardized acquisition and harmonization strategies. Furthermore, the use of the first treatment-day CBCT as the baseline reference may incorporate positioning variability and image noise relative to a conventional pre-treatment baseline. In addition, no formal assessment of inter- or intra-observer variability or deformable image registration accuracy was performed, and therefore the impact of contour uncertainty on radiomic feature stability cannot be fully excluded. Furthermore, no direct comparison with clinical or dosimetric baseline models was performed, and therefore the incremental predictive value of CBCT-derived radiomics relative to established predictors remains to be determined. Additionally, the use of logistic regression imposes linear decision boundaries; although nonlinear modeling approaches may capture more complex feature interactions, they may do so at the expense of interpretability. Finally, integration of radiomic predictors with dosimetric and clinical variables will likely be necessary to support robust risk stratification and enable eventual clinical implementation.

## 5 Conclusion

CBCT-based delta-radiomics of the supraglottic region demonstrates the potential to differentiate post-radiotherapy dysphagia in patients with HPV-positive oropharyngeal cancer. A ratio-to-baseline framework combined with weekly aggregation enabled longitudinal characterization of treatment-induced tissue change while maintaining statistical robustness. The predictive signal emerged early during treatment and was most coherently captured within the first four weeks, where parsimonious models achieved strong discrimination without increased dimensional complexity.

Extending temporal windows beyond Week 4 provided minimal incremental benefit relative to the added model complexity, underscoring the clinical relevance of early-phase imaging biomarkers. Importantly, feature stability was treated as contextual rather than exclusionary, acknowledging that dynamic biological processes may manifest as measurable temporal variability.

These findings support the potential of routinely acquired CBCT as a noninvasive, longitudinal biomarker platform for early dysphagia risk stratification and may facilitate future integration into adaptive radiotherapy paradigms, pending validation in larger, multi-institutional cohorts.

## Data Availability

The raw data supporting the conclusions of this article will be made available by the authors, without undue reservation.
