# Supplementary material for: Early toxicity prediction using longitudinal cone-beam CT delta-radiomics in HPV-positive oropharyngeal cancer
Source: Front Oncol. 2026 May 21;16:1830230. doi: 10.3389/fonc.2026.1830230 (PMC13233280; doi:10.3389/fonc.2026.1830230)

# Supplementary Materials

**Table S1.** Complete list of 42 IBSI-compliant radiomic features extracted from the supraglottic region. IBSI reference codes (third column) are provided for reproducibility.

| Feature Category | Feature Name (Abbreviation) | IBSI Code |
| --- | --- | --- |
| **Gray-Level Co-occurrence Matrix (GLCM)** |  |  |
|  | Contrast | ACUI |
|  | Correlation | NI2N |
|  | Dissimilarity | 8S9J |
|  | Energy | 8ZQL |
|  | Entropy | TU9B |
|  | Homogeneity | IB1Z |
|  | Sum Average | ZGXS |
|  | Variance | UR99 |
| **Gray-Level Run Length Matrix (GLRLM)** |  |  |
|  | Gray-Level Non-Uniformity (GLN) | R5YN |
|  | Gray-Level Variance (GLV) | 8CE5 |
|  | High Gray-Level Run Emphasis (HGRE) | G3QZ |
|  | Long Run Emphasis (LRE) | W4KF |
|  | Long Run High Gray-Level Emphasis (LRHGE) | 3KUM |
|  | Long Run Low Gray-Level Emphasis (LRLGE) | IVPO |
|  | Low Gray-Level Run Emphasis (LGRE) | V3SW |
|  | Run Length Variance (RLV) | SXLW |
|  | Run Percentage (RP) | 9ZK5 |
|  | Run-Length Non-Uniformity (RLN) | W92Y |
|  | Short Run Emphasis (SRE) | 220V |
|  | Short Run High Gray-Level Emphasis (SRHGE) | GD3A |
|  | Short Run Low Gray-Level Emphasis (SRLGE) | HTZT |
| **Gray-Level Intensity Histogram (First-order Statistics)** |  |  |
|  | Kurtosis | IPH6 |
|  | Skewness | KE2A |
|  | Variance | ECT3 |
| **Neighborhood Gray-Tone Difference Matrix (NGTDM)** |  |  |
|  | Busyness (BUSY) | NQ30 |
|  | Coarseness (COAR) | QCDE |
|  | Complexity (CPLX) | HDEZ |
|  | Contrast (CONT) | 65HE |
|  | Strength (STRG) | 1X9X |
| **Gray-Level Zone Size Matrix (GLZSM)** |  |  |
|  | Gray-Level Non-Uniformity (GLN) | JNSA |
|  | Gray-Level Variance (GLV) | BYLV |
|  | High Gray-Level Zone Emphasis (HGZE) | 5GN9 |
|  | Large Zone Emphasis (LZE) | 48P8 |
|  | Large Zones High Gray-Level Emphasis (LZHGE) | J17V |
|  | Large Zones Low Gray-Level Emphasis (LZLGE) | YH51 |
|  | Low Gray-Level Zone Emphasis (LGZE) | XMSY |
|  | Short Zone Emphasis (SZE) | 5QRC |
|  | Short Zones High Gray-Level Emphasis (SZHGE) | HW1V |
|  | Short Zones Low Gray-Level Emphasis (SZLGE) | 5RAI |
|  | Zone Percentage (ZP) | P30P |
|  | Zone Size Non-Uniformity (ZSN) | 4JP3 |
|  | Zone Size Variance (ZSV) | 3NSA |

All delta definitions, repeated random two-thirds resampling procedures (1000 iterations), and 95% percentile-based confidence interval calculations (2.5th–97.5th percentiles) are detailed in the main manuscript.

**Supplementary Figure S1.** Spearman correlation matrix of 43 ratio-to-baseline delta features. Correlation filtering threshold |ρ| > 0.8 was applied prior to Random Forest ranking.
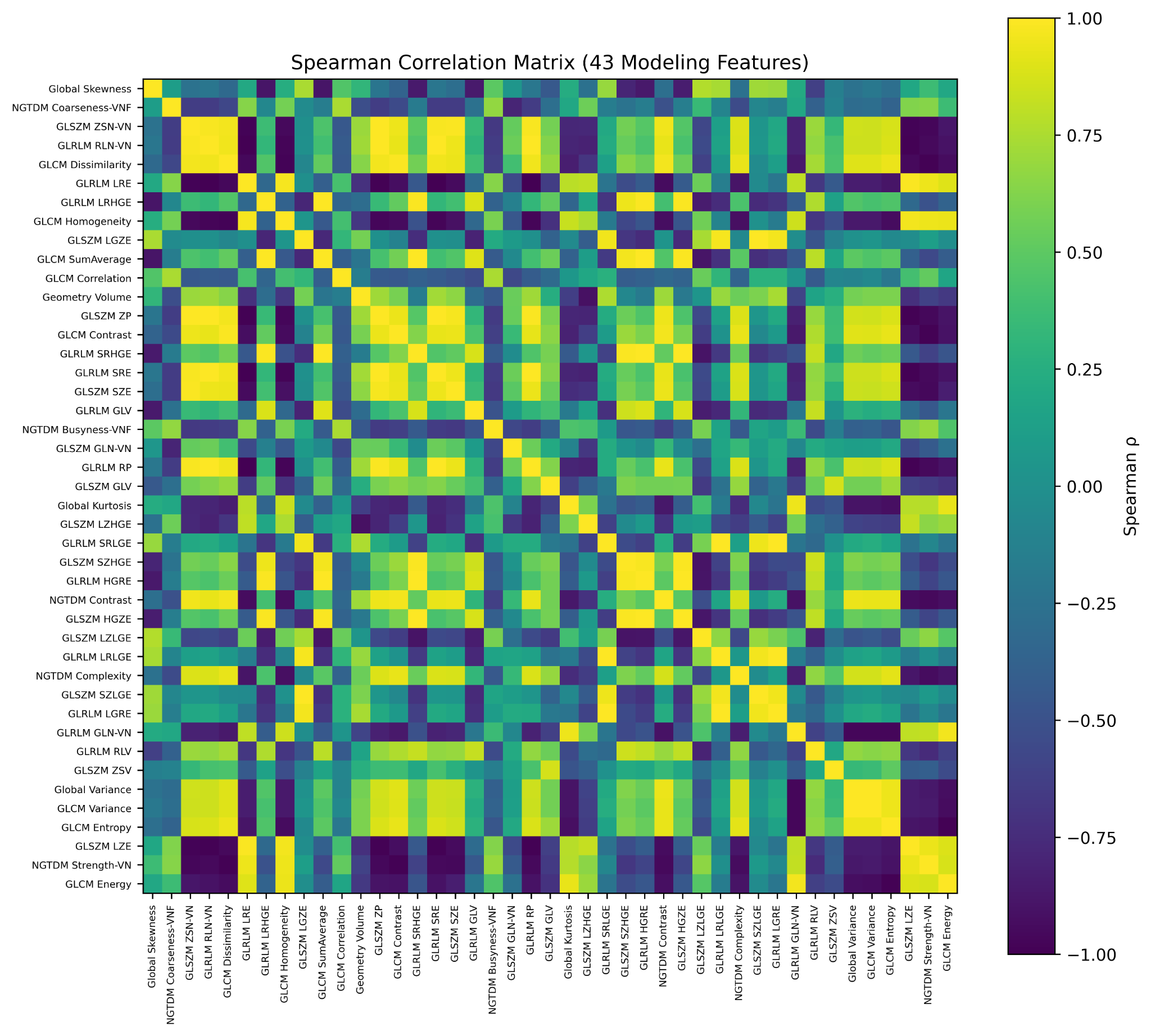


**Supplementary Figure S2.** Random Forest predictor ranking for the Week 2 temporal model using out-of-bag (OOB) permutation importance.


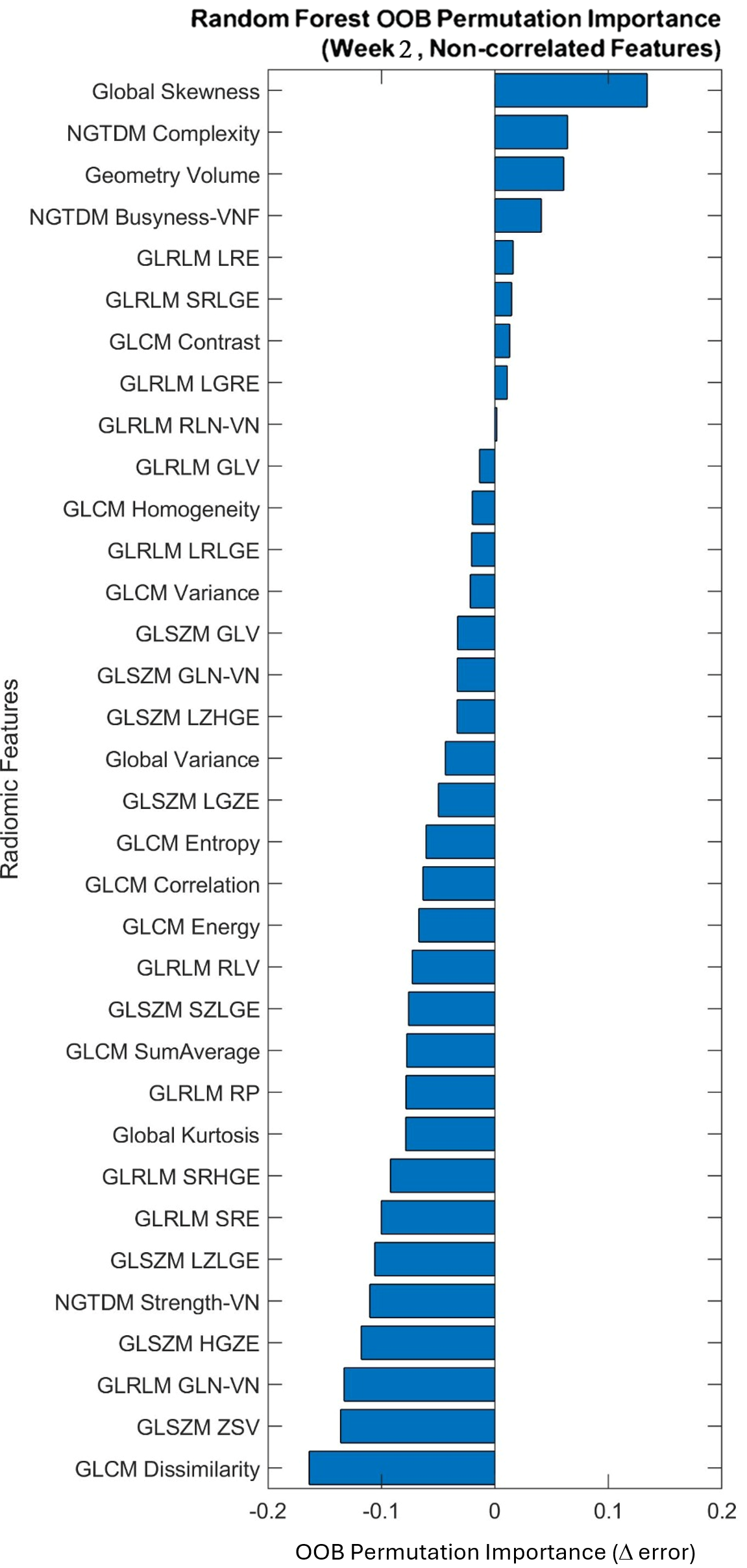


**Supplementary Figure S3.** Random Forest predictor ranking for the Weeks 1–6 temporal aggregation model using out-of-bag (OOB) permutation importance.


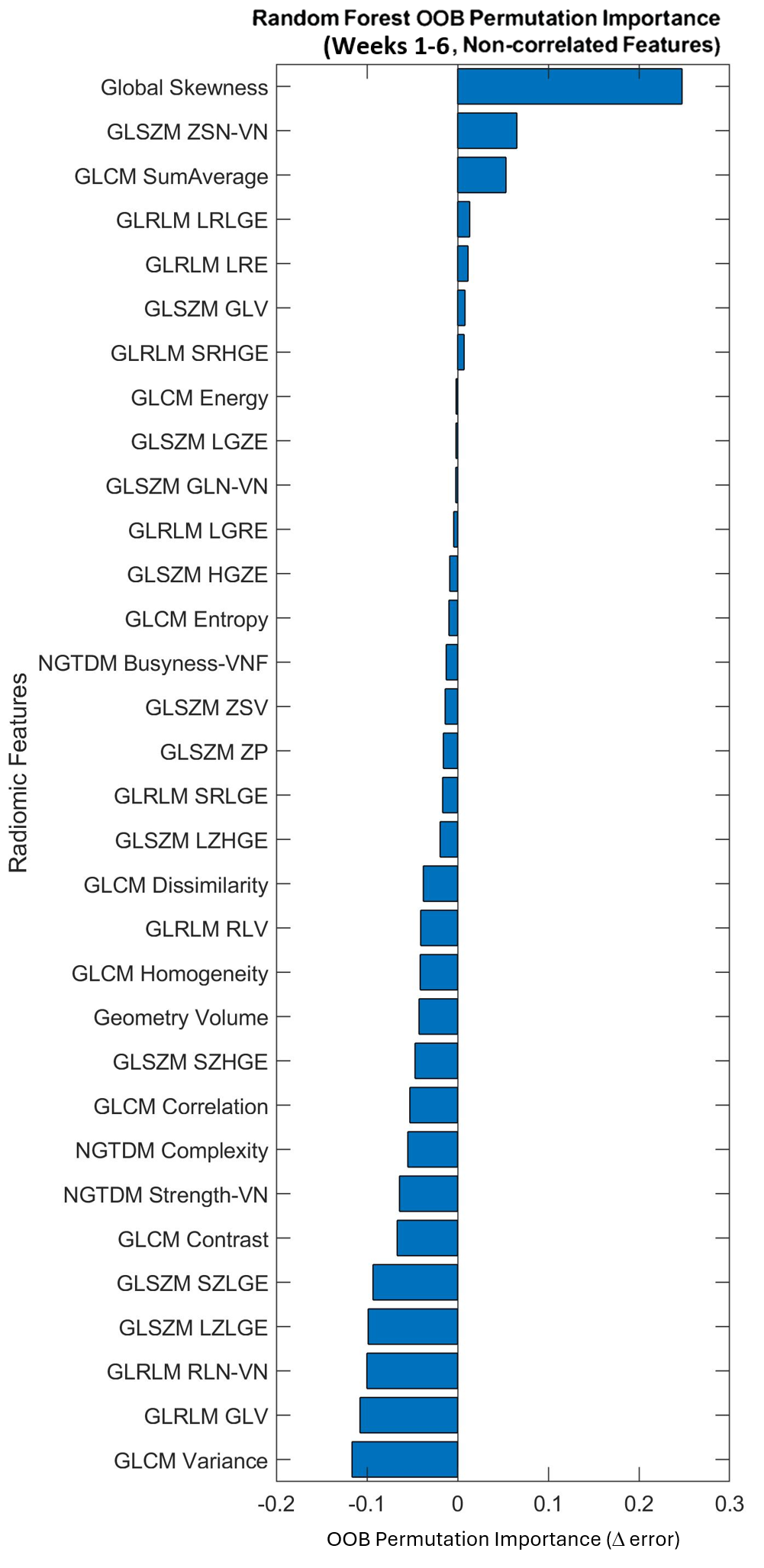


**Supplementary Figure S4.** AUC versus feature count for the Week 2 model. Error bars represent 95% percentile-based confidence intervals (2.5th–97.5th) derived from 1000 repeated two-thirds resampling iterations.


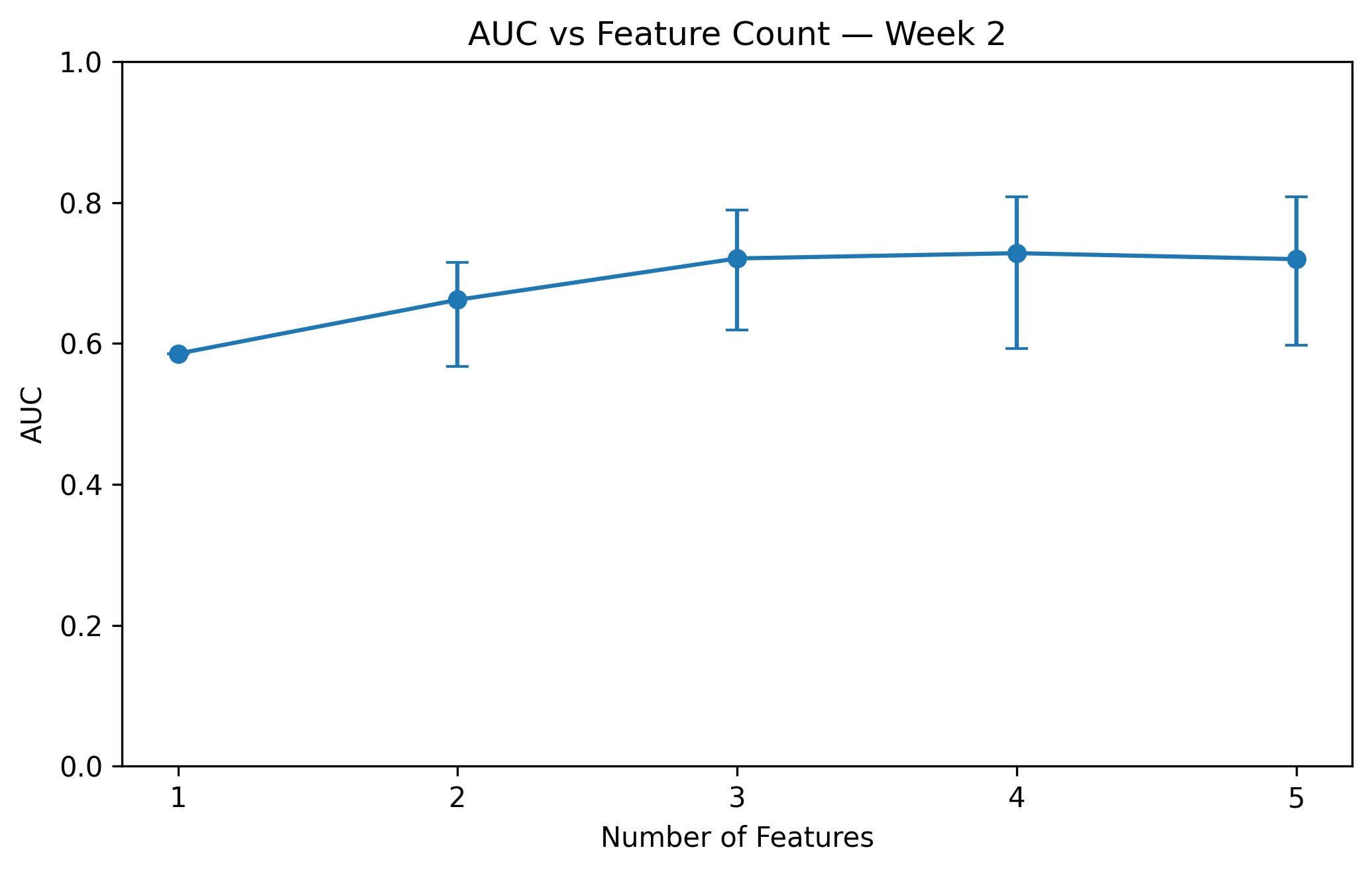


**Supplementary Figure S5.** AUC versus feature count for the Weeks 1–6 model. Error bars represent 95% percentile-based confidence intervals (2.5th–97.5th) derived from 1000 repeated two-thirds resampling iterations


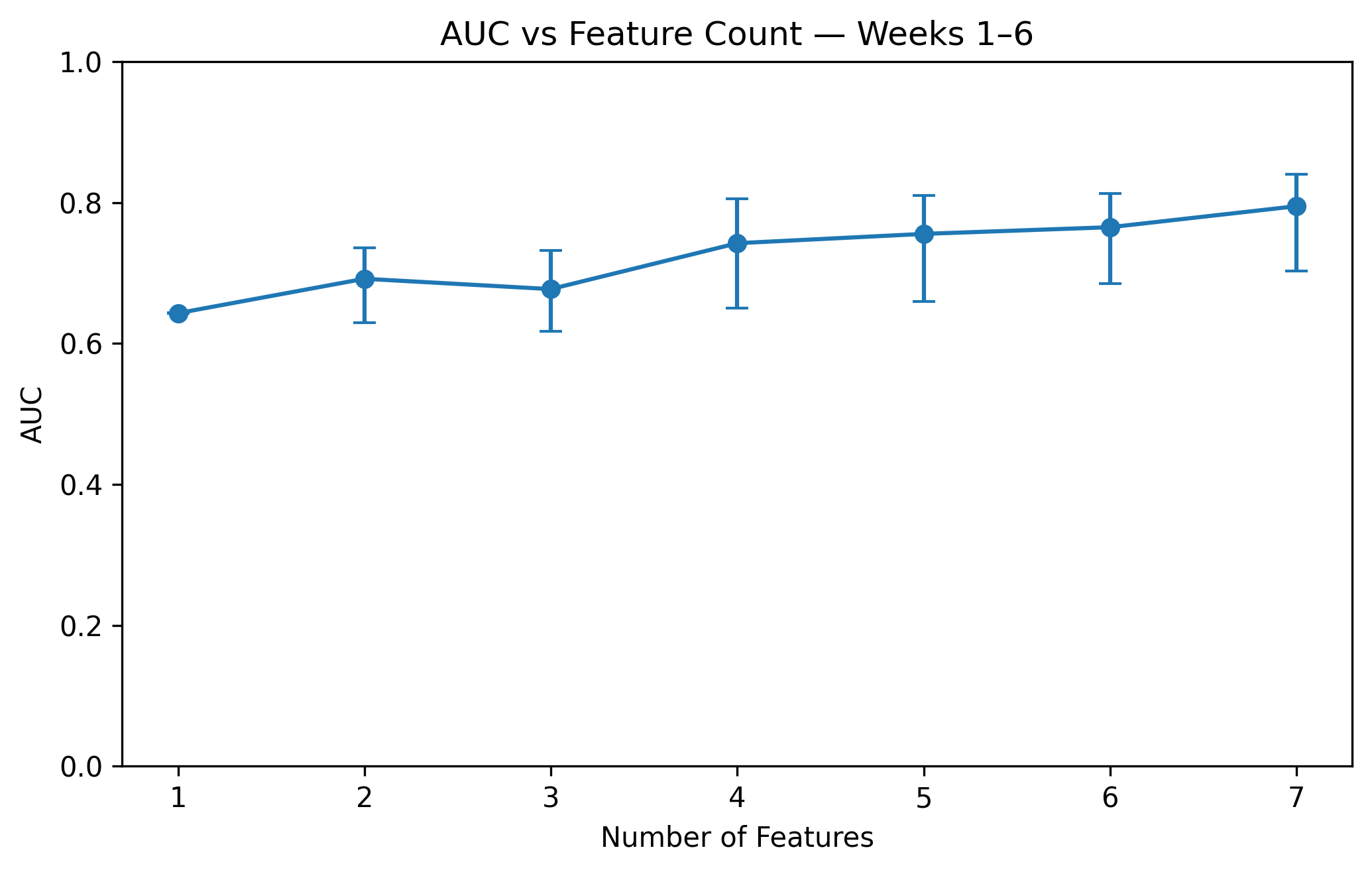


**Table S2.** Threshold-based performance metrics (sensitivity, specificity, positive predictive value [PPV], negative predictive value [NPV], and accuracy) for final models across evaluated temporal windows.

| Temporal Model | No. Features | AUC (95% CI) | Sensitivity | Specificity | PPV | NPV | Accuracy |
| --- | --- | --- | --- | --- | --- | --- | --- |
| Week 2 | 4 | 0.73 (0.60–0.81) | 0.75 | 0.74 | 0.63 | 0.83 | 0.74 |
| Weeks 1–4 | 3 | 0.78 (0.72–0.81) | 0.63 | 1.00 | 1.00 | 0.82 | 0.86 |
| Weeks 1–6 | 7 | 0.80 (0.71–0.84) | 0.88 | 0.74 | 0.67 | 0.91 | 0.79 |

**Supplementary Figure S6.** Distribution of ICC-like stability indices across ratio-to-baseline delta features.


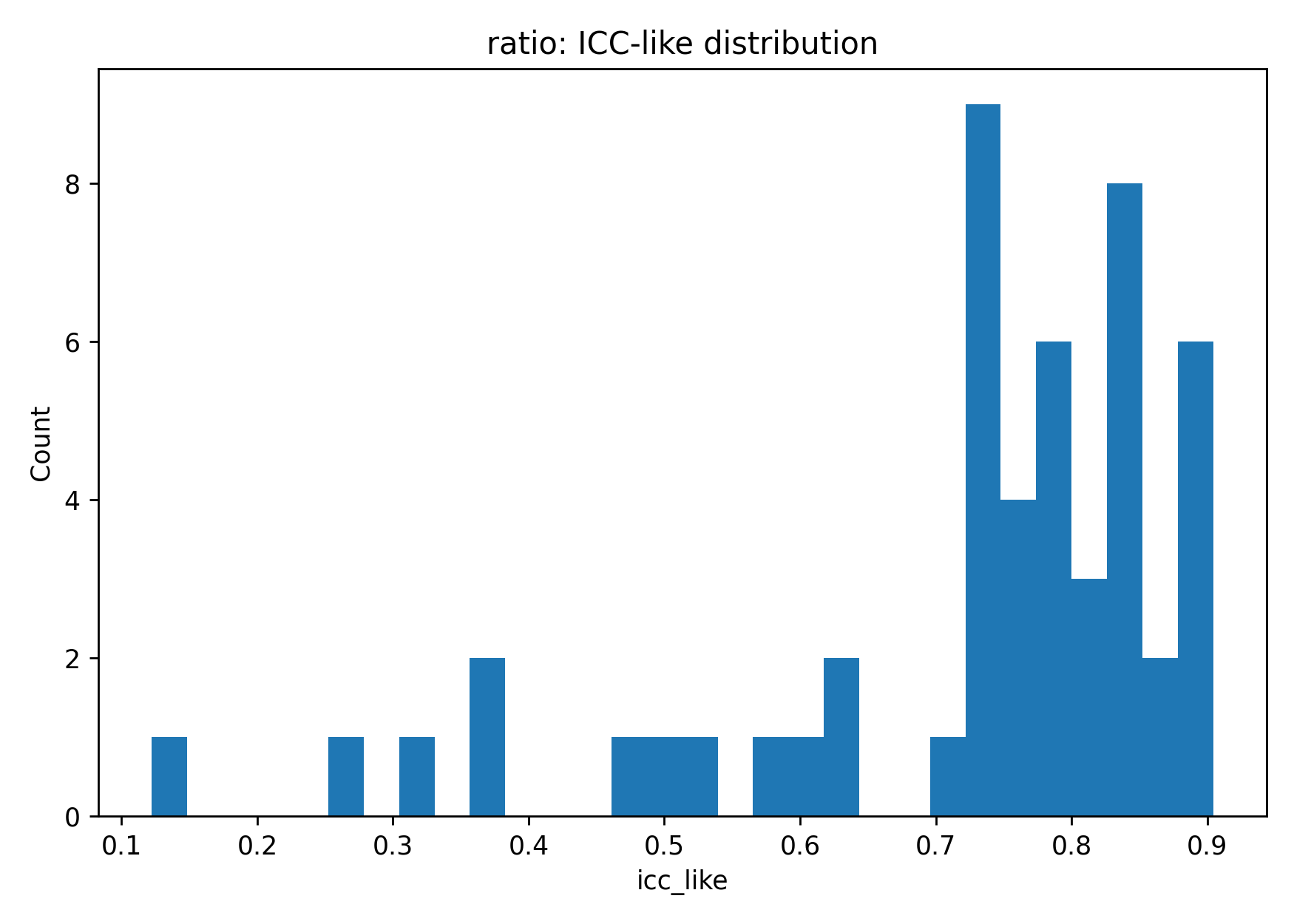


**Supplementary Figure S7.** Mean within-patient coefficient of variation (CV) across ratio-to-baseline delta features.


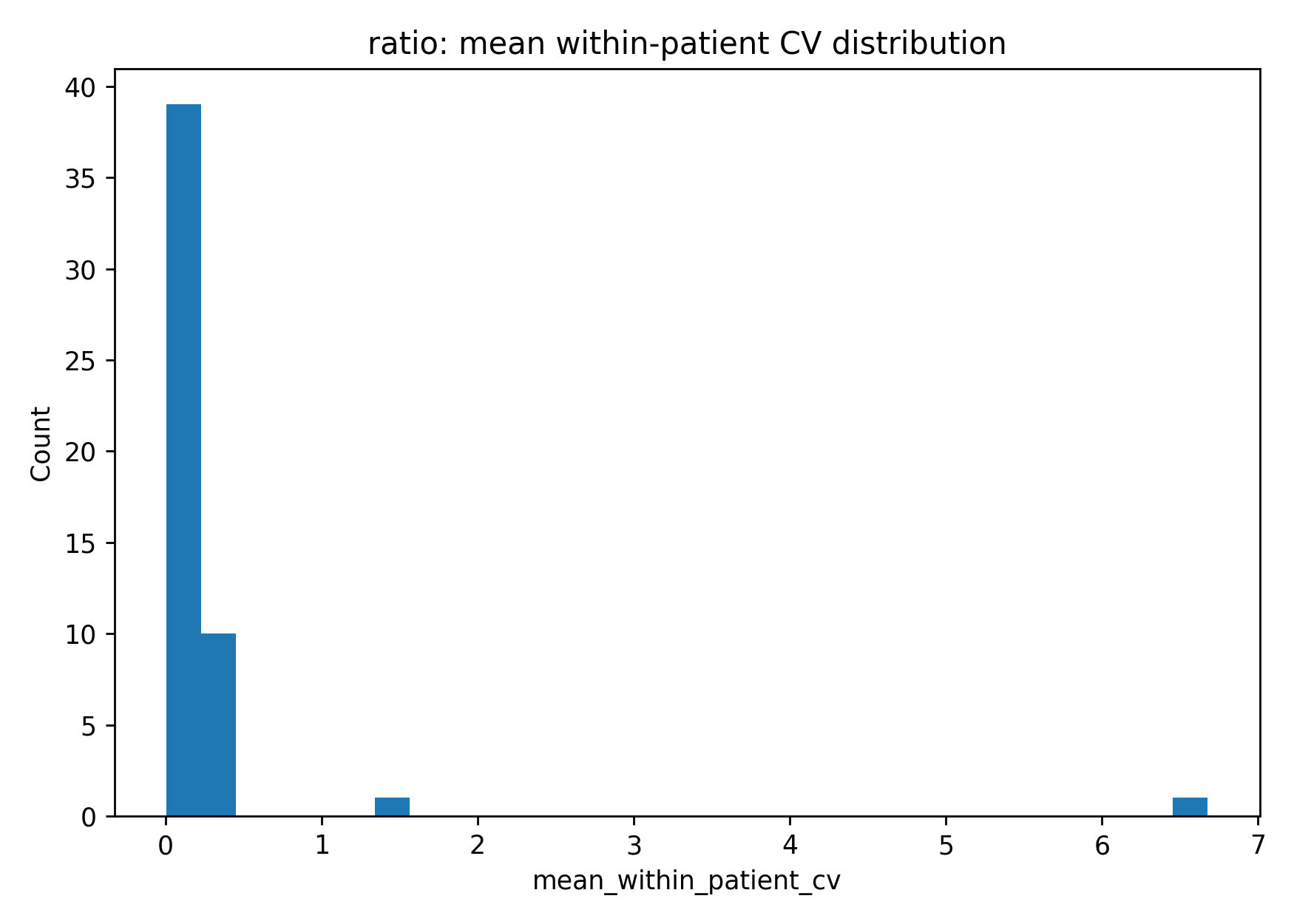


**Supplementary Figure S8.** Mean within-patient standard deviation (SD) across ratio-to-baseline delta features.


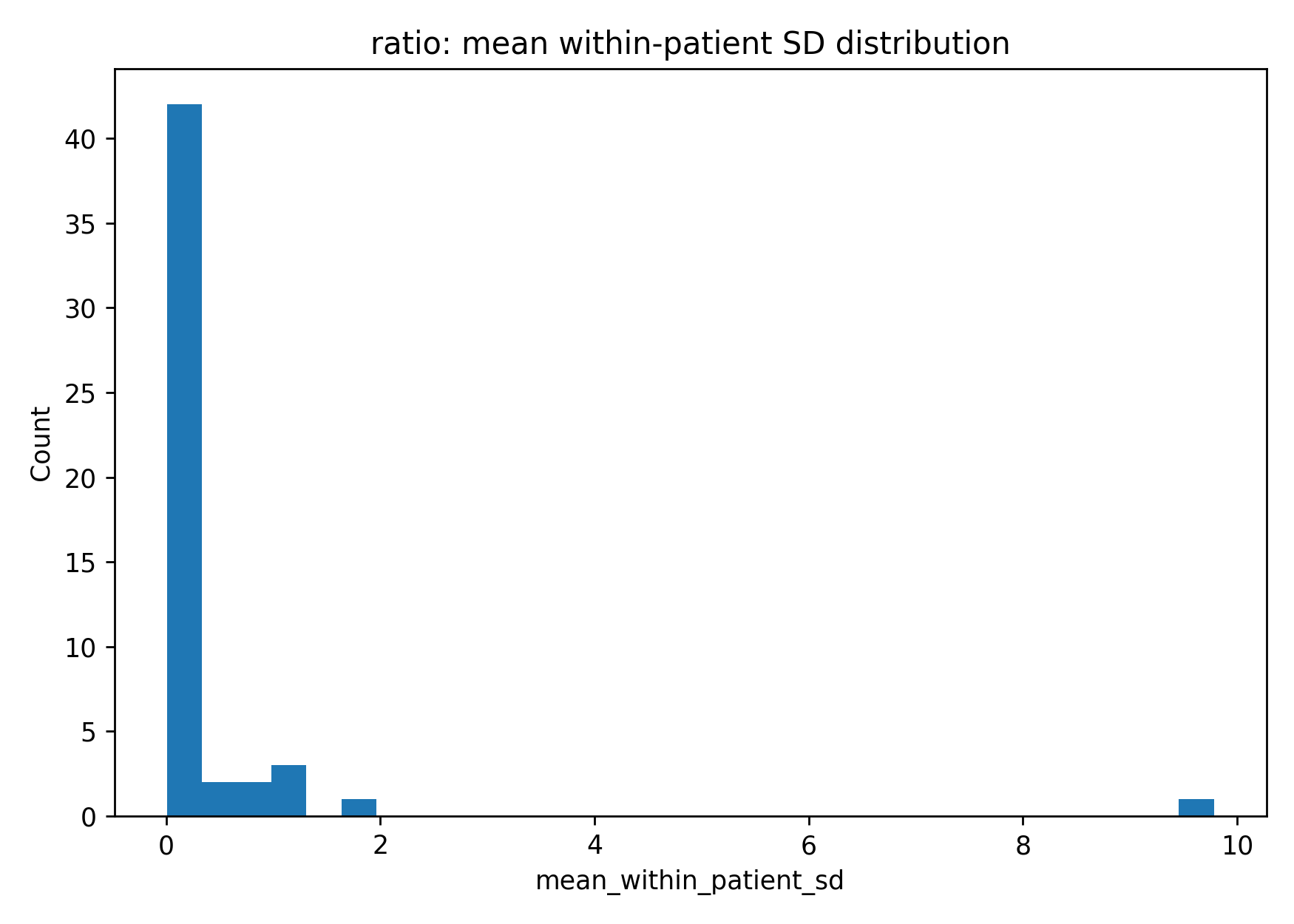


**Supplementary Figure S9.** Relationship between ICC-like stability index and within-patient coefficient of variation (CV) across delta features.


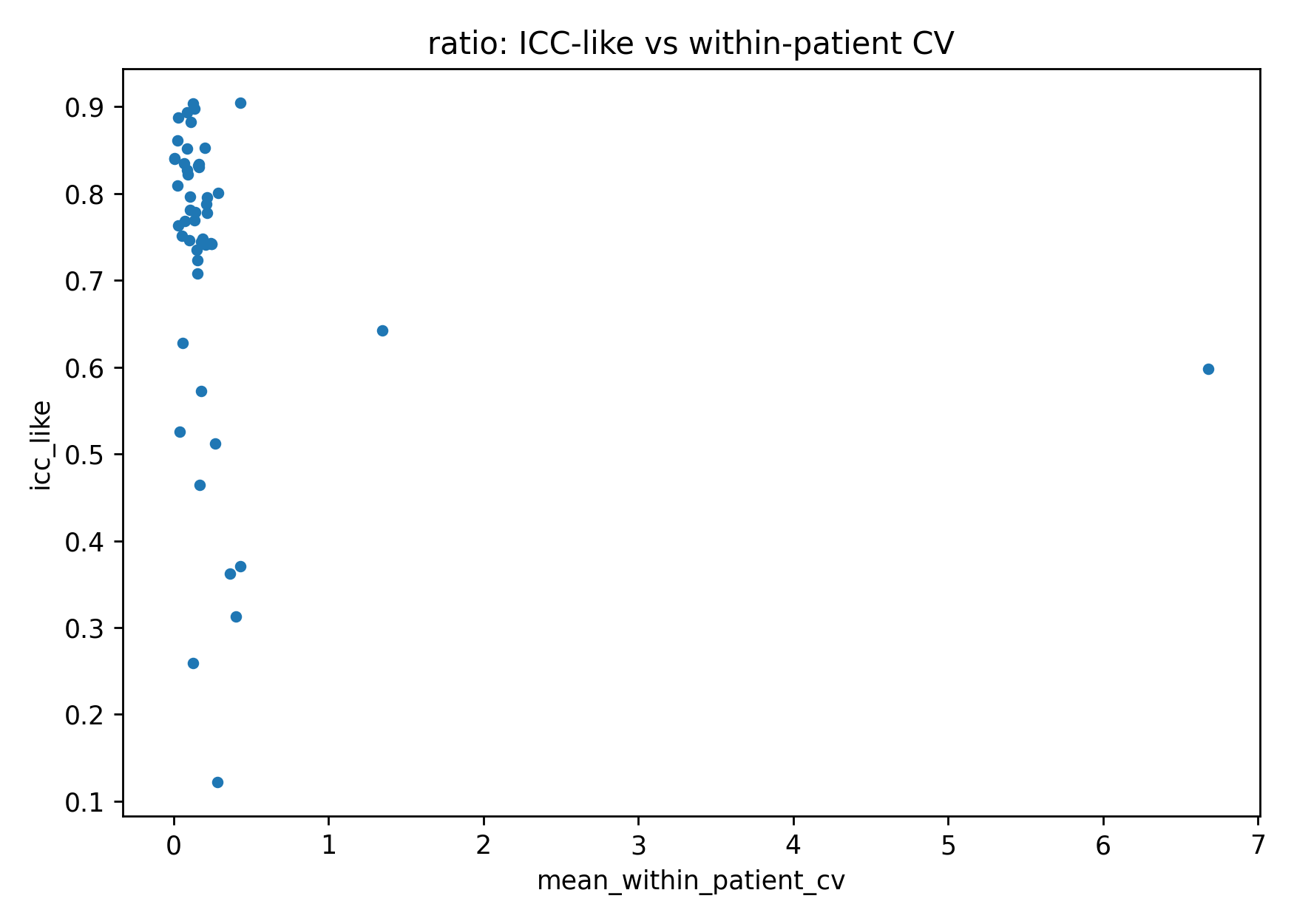

Supplement: Supplementary file 1 [file SupplementaryFile1.docx]
